# Supplementary material for: A Critical View of the Application of the APEX Software (Aqueous Photochemistry of Environmentally-Occurring Xenobiotics) to Predict Photoreaction Kinetics in Surface Freshwaters
Source: Molecules. 2019 Dec 18;25(1):9. doi: 10.3390/molecules25010009 (PMC7017383; doi:10.3390/molecules25010009)
Supplement: Supplementary file 1 [file molecules-25-00009-s001.zip › 3.2.4_gcc-4.4.0.zip!/3.2.4_gcc-4.4.0/3.2.4_gcc-4.4.0/doc/octave/html/interpreter/Multi_002ddimensional-Interpolation.html]

Multi-dimensional Interpolation - Untitled


Previous: One-dimensional Interpolation,
Up: Interpolation


---

### 28.2 Multi-dimensional Interpolation

There are three multi-dimensional interpolation functions in Octave, with
similar capabilities. Methods using Delaunay tessellation are described
in Interpolation on Scattered Data.

— Function File: zi = **interp2** (x, y, z, xi, yi)  
— Function File: zi = **interp2** (Z, xi, yi)  
— Function File: zi = **interp2** (Z, n)  
— Function File: zi = **interp2** (..., method)  
— Function File: zi = **interp2** (..., method, extrapval)  
> Two-dimensional interpolation. x, y and z describe a
> surface function. If x and y are vectors their length
> must correspondent to the size of z. x and y must be
> monotonic. If they are matrices they must have the `meshgrid`
> format.
>
> `interp2 (`x`,` y`,` Z`,` xi`,` yi`, ...)`: Returns a matrix corresponding to the points described by the matrices xi, yi. If the last argument is a string, the interpolation method can be specified. The method can be 'linear', 'nearest' or 'cubic'. If it is omitted 'linear' interpolation is assumed. `interp2 (`z`,` xi`,` yi`)`: Assumes x `= 1:rows (`z`)` and y `= 1:columns (`z`)` `interp2 (`z`,` n`)`: Interleaves the matrix z n-times. If n is omitted a value of n `= 1` is assumed.
>
> The variable method defines the method to use for the
> interpolation. It can take one of the following values
>
> 'nearest': Return the nearest neighbor. 'linear': Linear interpolation from nearest neighbors. 'pchip': Piece-wise cubic hermite interpolating polynomial (not implemented yet). 'cubic': Cubic interpolation from four nearest neighbors. 'spline': Cubic spline interpolation–smooth first and second derivatives throughout the curve.
>
> If a scalar value extrapval is defined as the final value, then
> values outside the mesh as set to this value. Note that in this case
> method must be defined as well. If extrapval is not
> defined then NA is assumed.
>
>
>
> **See also:** interp1.

— Function File: vi = **interp3** (x, y,z, v, xi, yi, zi)  
— Function File: vi = **interp3** (v, xi, yi, zi)  
— Function File: vi = **interp3** (v, m)  
— Function File: vi = **interp3** (v)  
— Function File: vi = **interp3** (..., method)  
— Function File: vi = **interp3** (..., method, extrapval)  
> Perform 3-dimensional interpolation. Each element of the 3-dimensional
> array v represents a value at a location given by the parameters
> x, y, and z. The parameters x, x, and
> z are either 3-dimensional arrays of the same size as the array
> v in the 'meshgrid' format or vectors. The parameters xi, etc.
> respect a similar format to x, etc., and they represent the points
> at which the array vi is interpolated.
>
> If x, y, z are omitted, they are assumed to be
> `x = 1 : size (`v`, 2)`, `y = 1 : size (`v`, 1)` and
> `z = 1 : size (`v`, 3)`. If m is specified, then
> the interpolation adds a point half way between each of the interpolation
> points. This process is performed m times. If only v is
> specified, then m is assumed to be `1`.
>
> Method is one of:
>
> 'nearest': Return the nearest neighbor. 'linear': Linear interpolation from nearest neighbors. 'cubic': Cubic interpolation from four nearest neighbors (not implemented yet). 'spline': Cubic spline interpolation–smooth first and second derivatives throughout the curve.
>
> The default method is 'linear'.
>
> If extrap is the string 'extrap', then extrapolate values beyond
> the endpoints. If extrap is a number, replace values beyond the
> endpoints with that number. If extrap is missing, assume NA.
>
>
>
> **See also:** interp1, interp2, spline, meshgrid.

— Function File: vi = **interpn** (x1, x2, ..., v, y1, y2, ...)  
— Function File: vi = **interpn** (v, y1, y2, ...)  
— Function File: vi = **interpn** (v, m)  
— Function File: vi = **interpn** (v)  
— Function File: vi = **interpn** (..., method)  
— Function File: vi = **interpn** (..., method, extrapval)  
> Perform n-dimensional interpolation, where n is at least two.
> Each element of the n-dimensional array v represents a value
> at a location given by the parameters x1, x2, ..., xn.
> The parameters x1, x2, ..., xn are either
> n-dimensional arrays of the same size as the array v in
> the 'ndgrid' format or vectors. The parameters y1, etc. respect a
> similar format to x1, etc., and they represent the points at which
> the array vi is interpolated.
>
> If x1, ..., xn are omitted, they are assumed to be
> `x1 = 1 : size (`v`, 1)`, etc. If m is specified, then
> the interpolation adds a point half way between each of the interpolation
> points. This process is performed m times. If only v is
> specified, then m is assumed to be `1`.
>
> Method is one of:
>
> 'nearest': Return the nearest neighbor. 'linear': Linear interpolation from nearest neighbors. 'cubic': Cubic interpolation from four nearest neighbors (not implemented yet). 'spline': Cubic spline interpolation–smooth first and second derivatives throughout the curve.
>
> The default method is 'linear'.
>
> If extrapval is the scalar value, use it to replace the values
> beyond the endpoints with that number. If extrapval is missing,
> assume NA.
>
>
>
> **See also:** interp1, interp2, spline, ndgrid.

A significant difference between `interpn` and the other two
multidimensional interpolation functions is the fashion in which the
dimensions are treated. For `interp2` and `interp3`, the 'y'
axis is considered to be the columns of the matrix, whereas the 'x'
axis corresponds to the rows of the array. As Octave indexes arrays in
column major order, the first dimension of any array is the columns, and
so `interpn` effectively reverses the 'x' and 'y' dimensions.
Consider the example

```
     x = y = z = -1:1;
     f = @(x,y,z) x.^2 - y - z.^2;
     [xx, yy, zz] = meshgrid (x, y, z);
     v = f (xx,yy,zz);
     xi = yi = zi = -1:0.1:1;
     [xxi, yyi, zzi] = meshgrid (xi, yi, zi);
     vi = interp3(x, y, z, v, xxi, yyi, zzi, 'spline');
     [xxi, yyi, zzi] = ndgrid (xi, yi, zi);
     vi2 = interpn(x, y, z, v, xxi, yyi, zzi, 'spline');
     mesh (zi, yi, squeeze (vi2(1,:,:)));
```

where `vi` and `vi2` are identical. The reversal of the
dimensions is treated in the `meshgrid` and `ndgrid` functions
respectively.

In additional the support function `bicubic` that underlies the
cubic interpolation of `interp2` function can be called directly.

— Function File: zi = **bicubic** (x, y, z, xi, yi, extrapval)  
> Return a matrix zi corresponding to the bicubic
> interpolations at xi and yi of the data supplied
> as x, y and z. Points outside the grid are set
> to extrapval.
>
> See http://wiki.woodpecker.org.cn/moin/Octave/Bicubic
> for further information.
>
>
>
> **See also:** interp2.
